# Factors associated with visual field defects of optic disc drusen

**DOI:** 10.1371/journal.pone.0196001

**Published:** 2018-04-30

**Authors:** Kyoung Min Lee, Se Joon Woo, Jeong-Min Hwang

**Affiliations:** 1 Department of Ophthalmology, Seoul Metropolitan Government Seoul National University Boramae Medical Center, Seoul, Korea; 2 Department of Ophthalmology, Seoul National University Bundang Hospital, Seongnam, Gyeonggi, Korea; Rigshospitalet Glostrup, DENMARK

## Abstract

**Purpose:**

To investigate the prevalence and risk factors for visual field defect in patients with optic disc drusen (ODD).

**Methods:**

We assessed the visual field status of patients with ODD whose diagnosis were confirmed by spectral-domain optical coherence tomography (SD-OCT). Visual field defects were classified as normal, enlarged blind spot, or other defects. ODD were classified into either type 1 (without hyperreflective border and heterogenic internal reflectance) or type 2 (with hyperreflective border and lower internal reflectance). The prevalence and risk factors for each visual field defect was analyzed using logistic regression analysis and classification and regression tree (CART) modeling.

**Results:**

Of the 40 eyes with ODD, 33 (83%) eyes were categorized as type 1 and 7 (17%) eyes were categorized as type 2 ODD. Regarding the visual field defects, 19 (48%) eyes showed normal visual field, 11 (28%) eyes showed enlarged blind spot, and 9 (24%) eyes showed other defects. The latter was more frequent in type 2 ODD (*P* = 0.001). Logistic regression analysis revealed that the factor associated with other defects was the thinning of the average retinal nerve fiber layer (RNFL) (per 10 μm decrease, OR = 3.436, *P* = 0.004), and the factor associated with enlarged blind spot was the height of ODD (per 100 μm increase, OR = 3.956, *P* = 0.023). CART modeling revealed that the average RNFL thickness lesser than 85.5 μm, and then the ODD height larger than 348 μm were the best split-up factors for predicting the type of visual field defects.

**Conclusions:**

In this study, one-quarter of ODD patients showed abnormal visual field defect other than enlarged blind spot. These other visual field defects appeared to be associated with the axonal loss in the eyes with type 2 ODD.

## Introduction

Optic disc drusen (ODD) are acellular hyaline deposits within the optic nerve head [1]. Superficial or visible ODD can easily be observed via funduscopic examination [1]. Conversely, buried ODD may require additional diagnostic tests, such as ultrasonography, to detect a signal of ODD [1]. Histologic study, however, showed that superficial or visible ODD are also covered by the transparent retinal tissue, i.e. they are also buried, and the calcified border is observable using funduscopy [2]. It means that the indexes of calcification, if they exist in plenty, can be observed through the transparent retinal tissue over the ODD. Therefore, it is reasonable to assume that there are various types of ODD and some of them show clear index of calcification, while others do not.

The introduction of spectral-domain optical coherence tomography (SD-OCT) marked a paradigm shift for the diagnosis of ODD. It became possible to image the acellular deposits in the optic nerve head with or without, a border of high reflectance of calcification [3–8]. Moreover, buried ODD without a highly reflective border have different characteristics compared with visible ODD or buried ODD with a highly reflective border [4, 7]. In this perspective, it is important to differentiate ODD base on border reflectivity as shown in SD-OCT images, and not depend only on funduscopic examination to determine their prognosis.

A highly reflective border of some ODD may suggest calcification during the evolution of ODD [2]. It also suggests axonal degeneration that contributes to a phenotypic difference. On the other hand, the accumulation of acellular deposits may further induce axonal distress. The visual field defect, as a complication of ODD, can be understood in this context [1, 9–11]. Interestingly, the prevalence of visual field defect varies greatly between studies: from 75% of patients showing no or minimal visual field defect [12] to 86% of patients showing visual field defects [9]. Such inconsistency may be attributed to the difference in the proportion of various types of ODD in each study population, since the type of ODD could greatly influence the prevalence of visual field defect [13]. Hence, we aimed to evaluate this complex relationship between the types of ODD, as classified by SD-OCT, and visual field defects.

## Methods

This retrospective study was approved by the Institutional Review Board of Seoul National University Bundang Hospital (SNUBH). Informed written consent requirement was waived due to the retrospective nature of this study. This study also adhered to the Declaration of Helsinki.

### Participants

Patients with ODD, who visited SNUBH Neuro-Ophthalmology Clinic between February 2013 and October 2016, were enrolled in this study. All patients were seen by one of physician (J.M.H.) at our institution. They underwent the same ophthalmologic examination, including best-corrected visual acuity (BCVA) measurement, intraocular pressure (IOP) measurement, dilated funduscopic examination, fundus photography (VX-10; Kowa OptiMed, Tokyo, Japan), SD-OCT (Spectralis, Heidelberg Engineering, Heidelberg, Germany), and standard automated perimetry (Humphrey Field Analyzer II 750, 24–2 or 30–2, Swedish interactive threshold algorithm, Carl Zeiss Meditec, Jena, Germany).

All participants met the following inclusion criteria: Age ≥ 8 years, BCVA ≥ 20/40, documentation of ODD on the SD-OCT images, and repeated automated perimetry tests that were spaced out at least 6 months apart. The exclusion criteria were as follows: Retinal or neurologic disease that could cause visual field defects, IOP > 21 mmHg, combined optic disc edema defined by the nasal retinal nerve fiber layer (RNFL) thickness of greater than 78.0 μm [3], inability to obtain a good-quality image (i.e., quality score>15) using SD-OCT in any section, and unreliable visual field tests (fixation loss>25%, or false-positive or false-negative error rates>25%). When both eyes were eligible, the eye with a larger ODD was selected for analysis.

### Diagnosis and classification of ODD using SD-OCT

The deep optic nerve head complex was imaged using SD-OCT via the enhanced depth imaging (EDI) technique. Details on OCT protocol for scanning ODD were provided in the previous studies [3, 4]. In brief, approximately 37 horizontal B-scan section images that cover the optic disc and the peripapillary area were obtained for each eye (the scan line distance being determined automatically by the instrument). For each B-scan image, one hundred OCT frames were averaged to obtain a high-resolution image. The value of corneal curvature was entered into the Spectralis OCT system prior to the scan to remove any magnification error.

**Fig 1:**
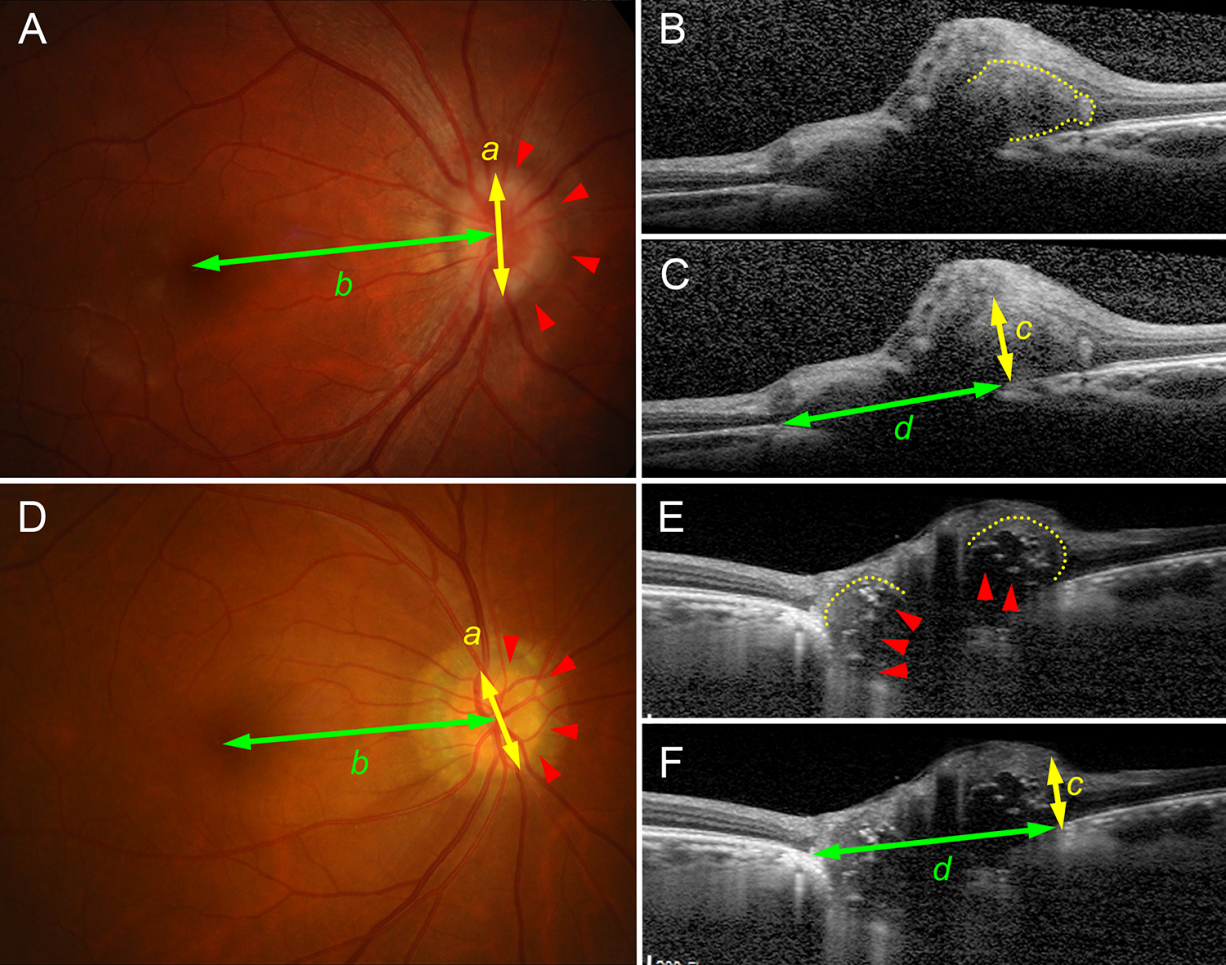
Measurements and classification of visual field defects. (**A**–**C**) Type 1 optic disc drusen (ODD). (**A**) Type 1 ODD creates a nasal halo (red arrowheads). The Relative Disc Index is defined as the ratio between the longest halo diameter (*a*) and foveo-disc diameter (*b*), and it represents the distortion induced by ODD on the enface plane. (**B**) B-scan spectral-domain optical coherence tomography (SD-OCT) image with demarcated border (yellow dotted line). (**C**) Same image of (**B**) with arrows indicative of the ODD height (*c*) and the Bruch’s membrane opening (BMO) diameter (*d*). The ODD height reflects the distortion induced by ODD in the axial plane. B-scan images with the largest ODD height and BMO diameter were used for their measurement, respectively. (**D**–**F**) Type 2 ODD. (**D**) The Relative Disc Index is defined in the same manner. Note the ODD are detectable through the funduscopic examination (red arrowheads) (**E**) B-scan SD-OCT image. Type 2 ODD composed of two portions: satellite lesions (red arrowheads) characterized by highly reflective border and low internal reflectance, and surrounding type 1 ODD-like deposits (yellow dotted lines). (**E**) Same image of (**F**) with arrows indicative of the ODD height (*c*) and the BMO diameter (*d*). The ODD height was measured from the most protruded part of the entire mass (satellite lesions and surrounding tissue) in the same manner.

ODD were diagnosed by visualizing the acellular deposits on B-scan SD-OCT images (Fig 1) [3–7]. ODD were defined as a mass with boundary, which contrasted to the normal optic nerve head anatomy; they did not belong to the normal retinal layers above the Bruch’s membrane opening (BMO) or they contrasted from the prelaminar tissue beneath the BMO. Eyes with comorbid optic nerve tumors were excluded [14].

ODD were classified into two types: type 1 included ODD without hyperreflective border and heterogenic internal reflectance (Fig 1A–1C), and type 2 included ODD with hyperreflective border and lower internal reflectance (Fig 1D–1F). Funduscopically visible ODD were type 2 ODD in all cases.

### Types of visual field defects

**Fig 2:**
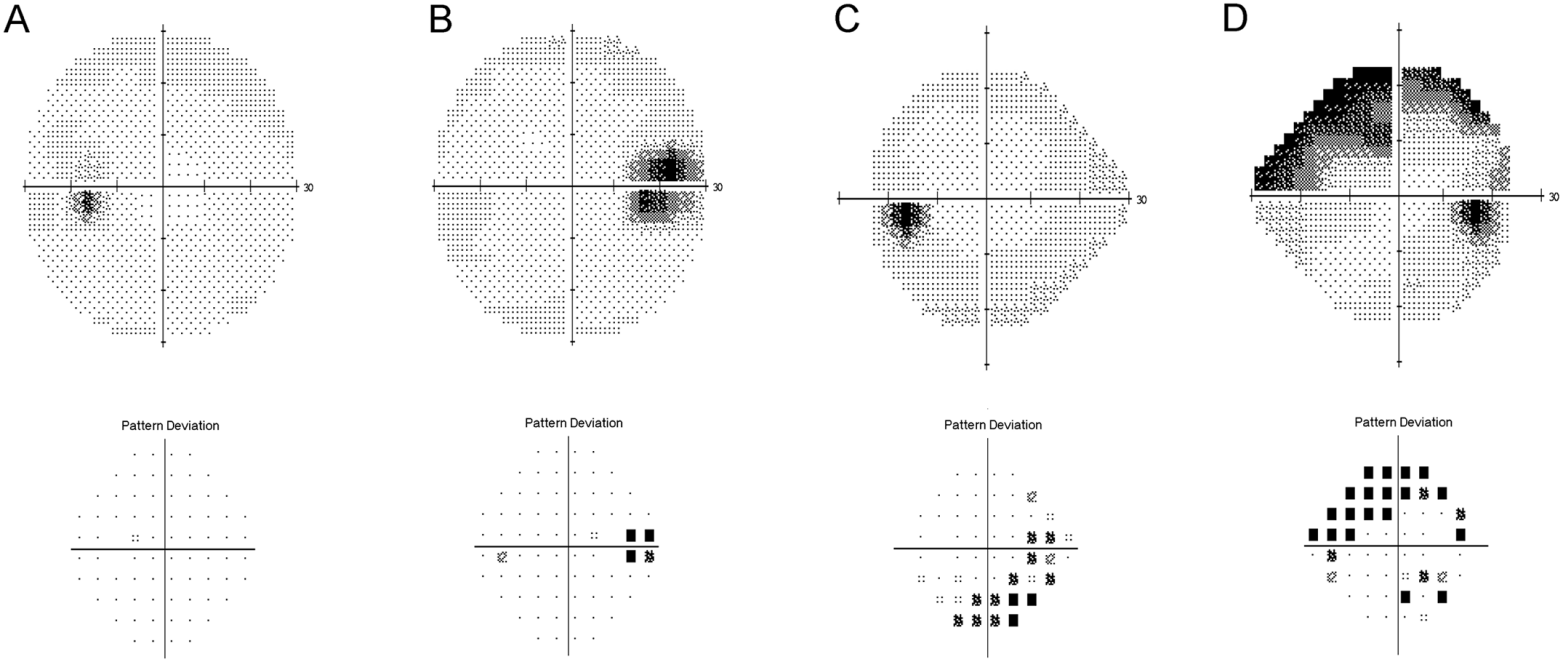
Types of visual field defects. (**A**) normal visual field, (**B**) enlarged blind spot, (**C**) other defects, and (**D**) bundle defect from the left respectively. The bundle defect is defined when other defects are connected to the blind spots. Please note that Humphrey visual field (HVF) test 30–2 strategy was used to ensure that no other defects were present in cases with normal visual field or enlarged blind spot.

Visual fields were classified into three categories (Fig 2): (1) normal, (2) enlarged blind spot, and (3) other defects. To be regarded as having visual field defects, visual field abnormalities must be seen in at least two separate visual field tests. Enlarged blind spot was defined as the presence of a point of probability of <0.5% next to the blind spot without any other defects (Fig 2B). In cases with normal visual field or enlarged blind spot, a repeat visual field test was performed using 30–2 strategy of Humphrey Visual Field (HVF) test to confirm the absence of peripheral defects. Other defects was defined as three contiguous non-edge points (allowing for two nasal-step edge points) with a probability of <5% being normal and one with a probability of <1% based on pattern deviation (Fig 2C). Other defects, which were connected to the blind spots, were subcategorized below the other defects as bundle defect (Fig 2D).

### Measurement of optic disc and ODD related parameters

Horizontal disc diameter was measured as the Bruch’s membrane opening (BMO) distance using the SD-OCT caliper tool. The B-scan SD-OCT image with the largest distance was used for the measurement (Fig 1, *d*).

Peripapillary distortion induced by ODD was measured in two ways. On one hand, the funduscopic distortion induced by ODD was measured as ‘Relative Disc Index.’ Relative Disc Index was calculated as the proportion between the longest diameter of the funduscopic disc margin and the fovea-to-disc diameter (Fig 1, *a*/*b*). Unlike type 2 ODD, the disc margin of type 1 ODD was not clearly discernible, due to the halo, as previously reported [15]. Halo was defined as a blurring of the optic disc margin on the fundus photographs [15]. In these cases, we measured the longest diameter of the halo (Fig 1A), since we wanted to measure the distortion induced by ODD, and not the actual disc size on the funduscopic plane. On the other hand, distortion induced by ODD along the axial plane was defined as the ‘ODD height’. The ODD height was measured as the perpendicular distance of protruded ODD above the BMO (Fig 1, *c*). The B-scan SD-OCT image with the largest height was used for the measurement. In cases with type 2 ODD, the height of protrusion was measured from the entire mass including satellite lesions (demarcated by the hyperreflective border and internal hyporeflectance, Fig 1E, red arrowheads) and surrounding granular tissue (Fig 1E, yellow dotted lines).

### Statistical analysis

The Kruskal-Wallis test and chi-square test were used to perform intergroup comparisons of continuous and categorical variables, respectively. Logistic regression analysis was used to determine the factors associated with each type of visual field defect. Classification and regression tree (CART) was implemented using 8 clinical variables which had been included in the logistic regression analysis in order to establish an explanatory model about the types of visual field defect [16]. The threshold for statistical significance was set at *P*<0.05. Statistical tests were performed using a commercially available software (Stata version 13.0, StataCorp, College Station, TX, USA) and R statistical packages version 3.3.3. Continuous data were presented as mean ± standard deviation, except where indicated otherwise.

## Results

Forty-seven eyes from 47 patients who were diagnosed with ODD and received at least 2 visual field tests were initially enrolled into this study. Of these, 4 eyes were excluded due to unreliable HVF test results, 2 eyes due to poor SD-OCT images, and 1 eye due to combined optic disc edema. These exclusions resulted in a final study population of 40 eyes from 40 patients.

Of the included 40 eyes, 33 (83%) eyes had type 1 ODD and 7 (17%) eyes had type 2 ODD. The demographic profiles of the study population are presented in Table 1. Patients with type 2 ODD, relative to those with type 1 ODD, were generally older in age (51.0±10.6 year vs 25.7±15.6 year, *P* = 0.003), had thinner average circumpapillary RNFL thickness (71±28 μm vs 102±21 μm, *P* = 0.004), and had worse visual field test results (mean deviation: –5.42±5.16 dB vs –1.76±2.32 dB, *P* = 0.084; pattern standard deviation: 5.96±3.21 dB vs 2.57±1.36 dB, *P* = 0.002) (Table 1).

**Table 1:** Patients’ characteristics by the types of optic disc drusen and visual field defects.

		ODD groups			Visual field groups			
	Total (*n* = 40)	Type 1 ODD (*n* = 33)	Type 2 ODD (*n* = 7)	*P*	Normal visual field (*n* = 19)	Enlarged blind spot (*n* = 12)	Other defects (*n* = 9)	*P*
Age (year)	30.1 (17.6)	25.7 (15.6)	51.0 (10.6)	0.003*	30.9 (17.6)	21.5 (13.0)	40.1 (18.9)	0.046^†^
Male:Female	19:21	15:18	4:3	0.574^‡^	9:10	5:7	5:4	0.820^‡^
Refractive errors (D)	–4.05 (3.08)	–4.45 (3.24)	–2.16 (0.82)	0.014*	–3.41 (1.65)	–3.89 (2.40)	–5.63 (5.34)	0.992^†^
IOP (mmHg)	11.6 (3.0)	11.7 (3.2)	10.7 (1.9)	0.390*	12.1 (3.4)	10.5 (2.9)	11.8 (1.8)	0.386^†^
MD (dB)	–2.40 (3.24)	–1.76 (2.32)	–5.42 (5.16)	0.084*	–0.87 (2.36)	–2.05 (1.43)	–6.11 (3.82)	0.001^†^
PSD (dB)	3.16 (2.19)	2.57 (1.36)	5.96 (3.21)	0.002*	1.65 (0.41)	4.22 (1.11)	4.95 (3.27)	<0.001^†^
RNFL thickness (μm)	97 (25)	102 (21)	71 (28)	0.004*	102 (9)	107 (34)	70 (17)	<0.001^†^
Visible ODD	7 (18%)	0	7 (100%)	n/a	0	2 (17%)	5 (56%)	0.001^‡^
ODD height (μm)	424 (106)	412 (108)	480 (75)	0.126*	369 (96)	496 (81)	443 (98)	0.005^†^
BMO diameter (μm)	1590 (220)	1569 (198)	1687 (302)	0.488*	1562 (161)	1698 (291)	1504 (181)	0.236^†^
Relative Disc Index	40 (5)	39 (4)	44 (5)	0.011*	38 (3)	42 (6)	39 (4)	0.143^†^

Mean values (standard deviations) are depicted.

*Comparisons were performed using independent *t*-test.

^†^Comparisons were performed using Kruskal-Wallis test.

^‡^Comparisons were performed using Chi-square test.

IOP = intraocular pressure; MD = mean deviation; PSD = pattern standard deviation; RNFL = retinal nerve fiber layer; ODD = optic disc drusen; BMO = bruch’s membrane opening.

According to the visual field test results, 19 (48%) eyes were considered as normal, 12 (30%) eyes as enlarged blind spot, and 9 (22%) eyes as other defects (Table 1). Both types of visual field defects were associated with larger ODD (normal vs enlarged blind spot vs other defects; 369±96 μm vs 496±81 μm vs 443±98 μm, *P* = 0.005, Table 1). Other defects were associated with thinner average circumpapillary RNFL thickness (normal vs enlarged blind spot vs other defects; 102±9 μm vs 107±34 μm vs 70±17 μm, *P*<0.001), and worse visual field test results (normal vs enlarged blind spot vs other defects; mean deviation: –0.87±2.36 dB vs –2.05±1.43 dB vs –6.11±3.82 dB, *P* = 0.001; pattern standard deviation: 1.65±0.41 dB vs 4.22±1.11 dB vs 4.95±3.27 dB, *P*<0.001, Table 1)

**Fig 3:**
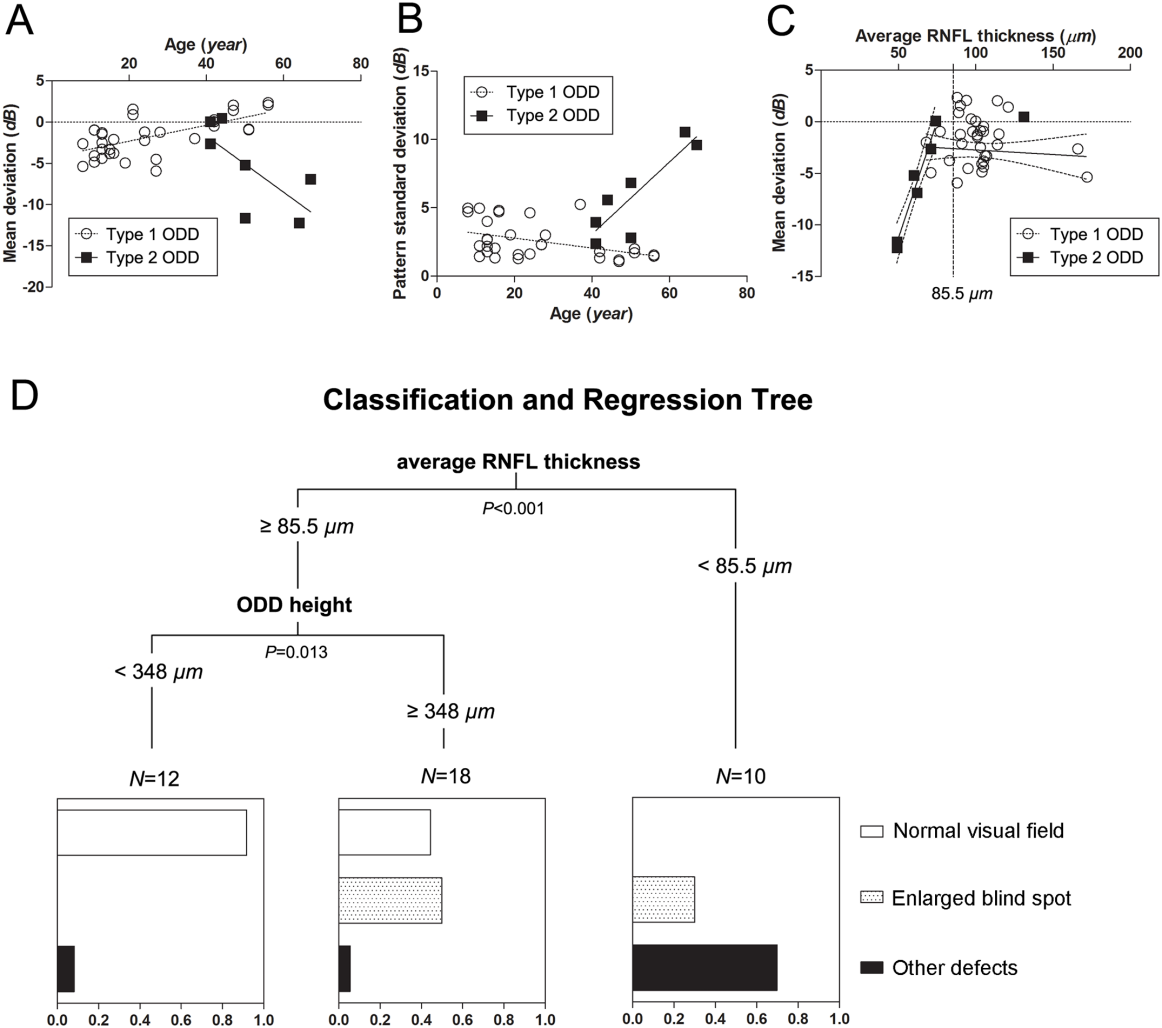
Scatter plot according to the type of ODD. (**A**) Age and mean deviation. The type 2 ODD group is older and has a worse mean deviation than the type 1 ODD group (Table 1). Age correlates negatively with the mean deviation in the type 2 ODD group (*r* = –0.764, *P* = 0.080), and positively in the type 1 ODD group (*r* = 0.648, *P*<0.001). (**B**) Age and pattern standard deviation. Similar correlations are observed both in the type 2 ODD group (*r* = 0.873, *P* = 0.010) and in the type 1 ODD group (*r* = –0.413, *P* = 0.017). (**C**) In the eyes with mean deviation values less than zero, the mean deviations are explained by RNFL thinning in the type 2 ODD group (*R*^*2*^ = 0.961, *P* = 0.001) and not in the type 1 ODD group (*R*^*2*^ = 0.016, *P* = 0.550). Regression lines and 95% prediction intervals are depicted. (**D**) A classification and regression tree (CART) model of associations between type of visual field defects and the following eight factors: age, sex, refractive error, intraocular pressure, the average circumpapillary RNFL thickness, the height of ODD, the diameter of Bruch’s membrane opening, and the Relative Disc Index. Plots are based on regression tree analyses, with each split based on the variable that best explains differences between groups. Comparisons were performed using Chi-square test in each node. Other defects are associated with the thinning of RNFL less than 85.5 μm, while enlarged blind spot is associated with the height of ODD larger than 348 μm. Please note the abrupt change of correlations between the mean deviations and RNFL thicknesses at the level of RNFL thickness of 85.5 μm (**C**).

The types of ODD affected the visual field defect differently (Fig 3). Type 2 ODD were associated with other defects (Table 1); other defects were observed in 5 eyes (71%) of type 2 ODD and in 4 eyes (12%) of type 1 ODD. Bundle defect, suggestive of RNFL loss, were observed in 4 out of the 9 eyes (44%) with other defects, and all of them had type 2 ODD. The mean deviations showed a negative correlation with age in the type 2 ODD group (*r* = –0.764, *P* = 0.080), while they showed a positive correlation with age in the type 1 ODD group (*r* = 0.648, *P*<0.001; Fig 3A). Similarly, pattern standard deviations showed a positive correlation with age in the type 2 ODD group (*r* = 0.873, *P* = 0.010), while they showed a negative correlation with age in the type 1 ODD group (*r* = –0.413, *P* = 0.017; Fig 3B). When patients with the mean deviation values of less than zero were analyzed separately, the mean deviation values were explained by the average circumpapillary RNFL thicknesses in the type 2 ODD group (*R*^*2*^ = 0.961, *P* = 0.001), while they were not explained that in the type 1 ODD group (*R*^*2*^ = 0.016, *P* = 0.550; Fig 3C).

Logistic regression analysis revealed that the average circumpapillary RNFL thickness was associated with other defects (per 10 μm decrease, OR = 3.346, *P* = 0.004; Table 2). Other factors related to the characteristics of patients (age, sex, refractive error and IOP), and the characteristics of anatomy of optic nerve head (ODD height, BMO diameter and Relative Disc Index) did not increase the risk of other defects (Table 2). Among the eyes without other defects (i.e., eyes that did not show scotoma, aside from enlarged blind spot), larger ODD height was determined to be the risk factor for enlarged blind spot (per 100 μm increase, OR = 3.956, *P* = 0.023; Table 2). The risk of enlarged blind spot was not increased by other factors (Table 2).

**Table 2:** Logistic regression analysis revealing the risk factors of each type of visual field defect.

	Other defects						Enlarged blind spot*					
	Univariate Analysis			Multivariate Analysis^†^			Univariate Analysis			Multivariate Analysis^†^		
	OR	*95% CI*	*P*	OR	*95% CI*	*P*	OR	*95% CI*	*P*	OR	*95% CI*	*P*
**Age**, *per 1 year older*	1.044	0.998–1.092	0.064	0.992	0.923–1.066	0.825	0.962	0.915–1.011	0.126			
**Female sex**	0.659	0.148–2.932	0.584				1.260	0.293–5.419	0.756			
**Refractive errors**, *per 1 diopter increse*	0.826	0.653–1.043	0.109				0.879	0.601–1.288	0.509			
**IOP**, *per 1mmHg increase*	1.034	0.805–1.329	0.793				0.848	0.663–1.084	0.187			
**RNFL thickness**, *per 10 μm decrease*	**3.300**	**1.538–7.092**	**0.002**	**3.436**	**1.475–8.000**	**0.004**	0.898	0.640–1.259	0.532			
**ODD height**, *per 100 μm increase*	1.253	0.614–2.557	0.536				**4.511**	**1.500–13.563**	**0.007**	**3.956**	**1.207–12.972**	**0.023**
**BMO diameter**, *per 100 μm increase*	0.756	0.501–1.143	0.185				1.351	0.921–1.983	0.124			
**Relative Disc Index**, *per 1 increase*	0.969	0.825–1.139	0.707				1.206	0.998–1.457	0.052	1.063	0.847–1.333	0.598

*Risk of having enlarged blind spot rather than the normal visual field among the patients without other defects.

^†^Variables with *P*<0.10 in the univariate analysis were included in the multivariate analysis.

Statistically significant values (*P*<0.05) are shown in bold.

OR = odds ratio; CI = confidence interval; IOP = intraocular pressure; RNFL = retinal nerve fiber layer; ODD = optic disc drusen; BMO = bruch’s membrane opening.

The CART analysis was carried out using the same variables to establish an explanatory model. The default tree was generated by allowing the program to determine the variable with the optimal first split. The results from the best tree indicated that the average circumpapillary RNFL thickness of lesser than 85.5 μm was the first split revealing the highest risk group of visual field defects (Fig 3D). After excluding that group, the height of ODD of larger than 348 μm was indicative of the higher risk of having visual field defects (Fig 3D).

## Discussion

**Fig 4:**
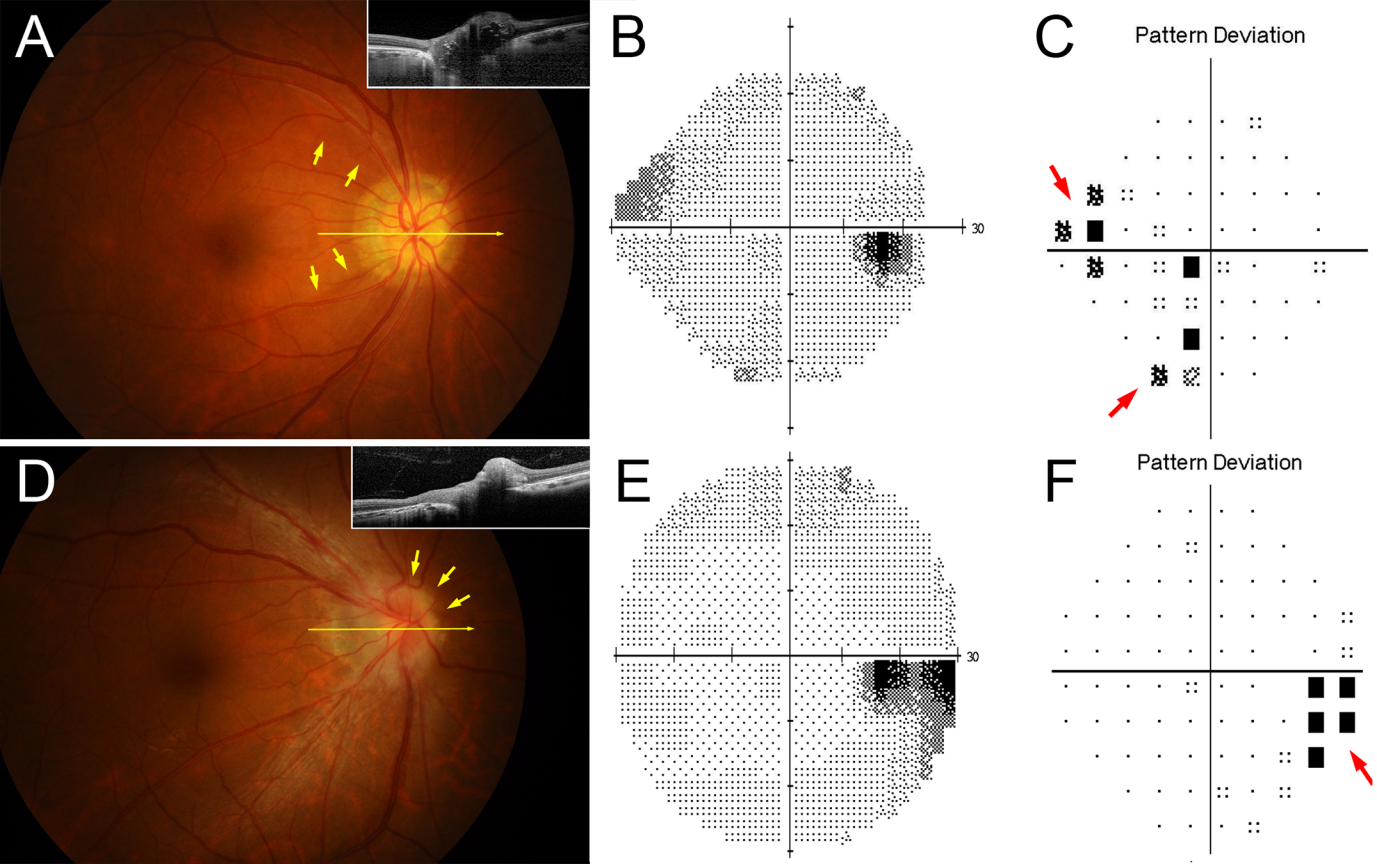
Sample cases of each type of visual field defect. (**A**–**C**) Other defects in the eye with type 2 ODD. (**A**) Fundus photography and SD-OCT image (inset). Highly reflective border is observed in the B-scan SD-OCT image, and ODD detectable through the funduscopic examination. A long arrow indicates where the SD-OCT scans. Short arrows point to the temporal margins of RNFL thinning. (**B**) Gray scale image of HVF. (**C**) Pattern deviation map shows superior nasal step and inferior defect (red arrows). corresponded to the areas of RNFL thinning in (**A**). Superior RNFL defect existed further from the foveo-disc axis (yellow arrows), thereby inferior visual field defect located further from the horizontal axis (red arrows). The location of visual field defect corresponded to the location of RNFL defect in the Garway-Heath map [22]. (**D**–**F**) Enlarged blind spot in the eye with type 1 ODD. (**D**) Fundus photography and SD-OCT image (inset). A long arrow indicates where the SD-OCT scans. Short arrows point to the superonasally located ODD. (**E**) Gray scale image of HVF. (**F**) Pattern deviation map shows the enlarged blind spot (red arrow). The enlargement proceeds in the inferotemporal direction, which is corresponded to the superonasal location of ODD.

According to the visual field test results, 48% of ODD eyes were normal, and 30% of ODD eyes showed enlarged blind spot. Visual field defects other than enlarged blind spot were found in 22% of the eyes, and 44% (4/9) of them had bundle defects, suggesting RNFL loss. In all cases with bundle defects, type 2 ODD was observed and RNFL defects existed along the corresponding locations of visual field defects (Fig 4A–4C).

Previous studies had revealed three types of visual field defects in patients with ODD: 1) arcuate defect, 2) enlargement of blind spot, and 3) concentric narrowing [9, 10, 13]. Axonal loss, induced by ODD, could lead to arcuate defect [9–11]. Arcuate defect was reported to be more frequent in adult patients [17] and in patients with visible ODD [9, 10]. In our study, other defects were exclusively associated with type 2 ODD, older age, and RNFL thinning. Moreover, bundle defect, which corresponded to arcuate defect, was found only in type 2 ODD with RNFL thinning. This suggests that arcuate defect with RNFL thinning may be associated with aging and type 2 ODD that are visible on funduscopy [12, 18–20].

Similar to a previous study using Goldmann perimetry [12], 75% of our patients did not show peripheral visual field defects. However, it is worth noting that many other studies reported higher rates of visual field defects compared to our study [9–11, 19, 21]. This disparity may be attributed to the differences in the study population: our patients were generally younger and fewer type 2 ODD patients were included in this study. Moreover, given that type 1 ODD, which can only be diagnosed using SD-OCT, will less likely have visual field defects, it explains the lower prevalence of visual field defects in this study. This speculation is supported by a recent report that clarified the association between ODD types and visual field defects [7].

Axonal loss induced by ODD could be measured as a decrease of circumpapillary RNFL thickness. This is a powerful marker in determining visual field deterioration (Fig 3C). The location of visual field defect corresponds to the location of RNFL defect matched by the Garway-Heath map [22] (Fig 4A–4C). Interestingly, RNFL thinning was observed only in the type 2 ODD group. The location of RNFL defect, however, was not associated with the location of type 2 ODD: ODD were located not only in the superotemporal and inferotemporal regions where RNFL defects appeared, but also in the nasal region (Fig 4A–4C). This discrepancy may favor the possibility that a common pathologic process in the type 2 ODD would result in both RNFL thinning and highly reflective border more than the possibility that type 2 ODD directly damage the RNFL and lead to its loss.

The main visual field defect of type 1 ODD was the enlargement of blind spot (Fig 4D–4F). Hoover et al. reported that this type of visual field defect was the most common type of all visual field defects in children with buried ODD [23]. In contrast to other defects, the visual field parameters (mean deviation and pattern standard deviation) did not correlate with RNFL thinning in the eyes with enlarged blind spot. Therefore, we speculate that the mechanism of the enlarged blind spot is not associated with axonal loss. We speculated that the association of younger age and poor visual parameters in the type 1 ODD may be the result of poor performance during visual field tests in younger subjects (Fig 2A and 2B).

To explain these complex interactions of axonal loss and other factors, we used the CART analysis based on binary recursive partitioning of data [16]. The CART analysis presents a decision tree that helps determine the most important variables in a data set. In our study, the CART analysis visualized the hierarchy of factors affecting visual field defects: firstly, the thinning of RNFL was the best predictor of defect elsewhere, and the ODD height was associated with enlarged blind spot thereafter (Fig 3D).

ODD can impede visual function [24, 25]. Possible mechanisms are 1) blockage of axonal transport [2, 26], 2) direct mechanical pressure [26, 27], and 3) circulatory disturbance [28, 29]. The average circumpapillary RNFL thickness could be the result of axonal loss, explaining the visual field deterioration well (Fig 3C). The RNFL thinning was observed exclusively in the type 2 ODD group. This suggested the highly reflective border of type 2 ODD might be associated with calcification related to the axonal death. After exclusion of the effect of RNFL thinning, the height of ODD associated protrusion was associated with the presence of an enlarged blind spot (Table 2). The association between ODD sizes and visual field defects was previously reported [7, 8, 30]. We suspect that the direct effect of ODD on the optic nerve axon, such as blockage of axonal transport or circulatory disturbance, may be responsible for the decrease in visual sensitivity near the blind spot.

It should be noted that we do not know whether type 1 ODD and type 2 ODD are in the same disease spectrum or they are totally different disease entities. Some argued that peripapillary hyperreflective ovoid mass-like structures (PHOMS) is a sign of axonal herniation and it should not be diagnosed as ODD [31, 32]. We speculated that some of type 1 ODD (or PHOMS) could turn into type 2 ODD by acquiring calcification for two reasons. First, in type 2 ODD, the lesions demarcated by a highly-reflective border have been shown to be surrounded by type 1 ODD-like deposits [4, 6, 7]. Type 1 ODD (or PHOMS) might indicate the axonal stress within the optic nerve head before the axonal death and calcification. Second, we had reported previously that the auto-fluorescent signal within type 1 ODD before ODD was visible on funduscopy [33]. In this context, type 1 ODD would have an important clinical implication since it would be more difficult to differentiate from optic disc edema than type 2 ODD, and it could turn into type 2 ODD with aging [34]. That is why we had included both type 1 and type 2 ODD in this study. Nevertheless, it is important to note that our measurements (such as the Relative Disc Index or ODD height) may have some limitations because they have different implications in different types of ODD.

This study has some limitations. First, as we stated above, there is an ongoing debate whether type 1 ODD (or PHOMS) are ODD [34] or not [31, 32]. We, however, believe that type 1 ODD are still important, or even more important than type 2 ODD, as one of differential diagnosis of optic disc edema [34]. Second, the peripheral visual field examinations were not performed. Although, all subjects with normal visual field or enlarged blind spot in our study did not complain about the visual field constriction and had underwent HVF 30–2 for confirmation, we were unable to assess whether these patients had peripheral visual field defects [35]. Third, the progression of visual field defect was not determined in this study. Although all repeated visual field examinations were spaced apart by at least six months in all subjects, a six-month duration may have been too short to properly determine the progression. Lastly, we were unable to assess the fluctuation of visual field defects. Occasionally, patients with ODD complained about transient obscuration of the visual field. In cases with hemorrhagic complications, it is possible for visual field defects to subside with time [36], or there might be other causes of fluctuations of visual field test results [28, 29]. Since only the repeated defect was defined as a visual field defect, it is possible that we overlooked the transient defects in this study.

In conclusion, 75% of our patients with ODD showed normal visual field or only enlarged blind spot. Enlarged blind spot was associated with a large protrusion induced by ODD. However, 25% of patients with ODD showed visual field defects other than enlarged blind spot, and they were associated with RNFL thinning and type 2 ODD. Therefore, visual field defects should be suspected and checked in patients with type 2 ODD which are highly suggestive of axonal loss.

## Supporting information

S1 File Optic disc drusen and visual field data set. (XLS)
